# A Real-Time PCR Assay for Detecting Codling Moth *Cydia pomonella* on Material Intercepted at U.S. Ports of Entry—A Valuable Tool for Specimen Identification

**DOI:** 10.3390/ijms26020707

**Published:** 2025-01-15

**Authors:** Alicia E. Timm, Luke R. Tembrock, Frida A. Zink, Kayla A. Mollet

**Affiliations:** 1 Department of Agricultural Biology, Colorado State University, Fort Collins, CO 80523-1177, USA; frida.zink@colostate.edu (F.A.Z.); kayla.mollet@colostate.edu (K.A.M.); 2 USDA-APHIS-PPQ-Science & Technology, Fort Collins, CO 80526, USA

**Keywords:** invasive species, Tortricidae, molecular identification, insect identification

## Abstract

Codling moth *Cydia pomonella* is well established nearly everywhere apples are grown. Due to this almost global distribution, larvae are often intercepted at U.S. ports of entry where immature specimens cannot be identified accurately to species leading to unnecessary quarantine actions. To assist with identifying intercepted *C. pomonella* from port inspections, we developed a probe-based real-time PCR assay to amplify the internal transcribed spacer (ITS) region 2 of *C. pomonella*. The assay was tested for inclusivity using 110 *C. pomonella* specimens from six continents. Analytical specificity was examined by testing related species intercepted at U.S. ports of entry, as well as non-targets with the same geographic distribution and host species as *C. pomonella*. The assay developed here identified all *C. pomonella* individuals correctly and produced appropriately negative results for all non-target species. These results ensure that the assay provides a rapid and accurate tool for unambiguously identifying *C. pomonella* among material intercepted at U.S. ports of entry. Since *C. pomonella* is not actionable, the ability to identify all life stages of *C. pomonella* conclusively will save time, effort, and money while also directing identification efforts towards species of current quarantine concern.

## 1. Introduction

Imported commodities are visually inspected by plant protection authorities to prevent the unintentional establishment of nonnative species in the United States. This process requires the identification, usually to species, of all insects intercepted at ports of entry. If authoritative identification is not possible, or identifications are possible only to higher taxonomic levels (e.g., family or genus), quarantine actions are justified. These actions may include phytosanitary measures such as fumigation or heat treatment of commodities [1]. Shipments may also be rejected, resulting in the possibly needless destruction of produce, while in extreme cases export actions of specific exporters may be reduced [2]. To prevent needless quarantine actions, accurate and straightforward identification of frequently intercepted species is essential.

The codling moth, *Cydia pomonella* L. (Lepidoptera: Tortricidae) is one of the most economically important apple pests worldwide, with the potential to cause complete crop devastation in apple orchards [3]. Adaptive behaviors such as multiple generations per breeding season, facultative diapause, and a host range including species in Rosaceae (apples, pears, peaches, plum, apricot, almond, and quince), Juglandaceae (walnut), Fabaceae (jack bean), Fagaceae (chestnut), Rutaceae (citrus), and Poaceae (maize), have allowed the insect to adapt to diverse climates and environments [4]. *Cydia pomonella* individuals are able to spread long distances through international trade of infested fruit, seeds, and other plant parts, as well as in packing material. These factors have resulted in *C. pomonella* being one of the most successful insect pest species worldwide in terms of invasiveness. *Cydia pomonella* is thought to have originated in south-eastern Europe or Asia. Over the last two centuries, this species has spread to all continents, with the exception of Antarctica, and is present in nearly 80 countries, including almost every country where apples are grown [5]. Although *C. pomonella* is notably absent in Japan and South Korea and has a limited distribution in Canada and parts of South America, its distribution may continue to expand as a result of climatic warming [5,6]. *Cydia pomonella* is well established in the U.S., where it is the main pest of apples. Even so, it is often intercepted at U.S. ports of entry due to its global distribution.

Morphological identification is the primary tool for identifying *C. pomonella* at U.S. ports. Most interceptions of internal feeding insects such as *C. pomonella* occur as larvae. Although larvae are the most common stage requiring accurate identification, they are also the most challenging stage to identify. This is particularly true of earlier larval instars, since the morphological characters necessary for identification cannot be clearly seen, with the same problem for eggs and pupae [7,8,9]. Several morphological keys are available for identifying tortricid larvae, including Nearctic fauna (e.g., [10,11,12]). Keys for identifying tortricid larvae frequently intercepted on *Castanea* [13] as well as other tortricid larvae frequently intercepted at U.S. ports of entry, including *C. pomonella* ([14], https://idtools.org/id/lepintercept/pomonella.html, accessed on 22 March 2024), have been developed. Despite these tools, larvae can be highly variable, and many specimens cannot be identified to species convincingly [14]. Using morphology, larval specimens of *C. pomonella* are frequently identified only to genus or family by port inspectors. Adult *C. pomonella* individuals are rarely intercepted but, in contrast to larvae, are more easily identified by morphological characters, including genitalic dissections [15].

The inability to accurately identify *C. pomonella* larval specimens, despite the species already being established in the U.S., is time-consuming and depletes scarce specialist resources. Such misidentifications may also have resulted in unnecessary quarantine actions. Consequently, there is a need to identify immature stages of *C. pomonella* rapidly and easily to support quarantine decisions at U.S. ports of entry by rapidly eliminating from consideration a commonly intercepted species that is already well established in North America. Such efforts will improve efficiency for specialists and allow them more time to focus on species of quarantine concern.

Molecular methods are increasingly being used to support insect identification and provide increased taxonomic resolution, particularly where insects are intercepted as immatures that cannot be identified using traditional morphological techniques. DNA barcoding, based on amplifying a ~650 bp segment of the mitochondrial cytochrome c oxidase I gene [16,17], has been widely used to identify insect species and has been found to be accurate in identifying known species, including *C. pomonella* [18] although instances in which DNA barcodes have limited discriminatory power have been noted [19]. The use of mitochondrial DNA (mtDNA) is associated with problems such as small effective population sizes, single-parent inheritance, heteroplasmy, and challenging thermodynamic properties based on nucleotide proportions [20,21]. In addition to these problems, DNA barcoding may take days to generate results, which means that it is not suitable for the identification of specimens that require urgent identification. In a number of cases spacer regions within the nuclear ribosomal DNA (rDNA) have been employed for insect molecular diagnostics as a substitute to the CO1 barcode as rDNA loci can be less susceptible to some of the problems found in mtDNA noted above (e.g., [22,23,24]). Several molecular assays have been developed to specifically identify *C. pomonella* (see Table 1), all targeting the same mtDNA barcode region. Barcenas et al. [7] developed diagnostic primers to discriminate *C. pomonella*, *Aspila molesta*, *A. prunivora*, and *A. packardi*, which all feed on apples in the U.S., using conventional and real-time PCR. This assay was adapted by Amano and Higo [25], who developed diagnostic primers to discriminate *C. pomonella* from other tortricid pests feeding on cherries in North America. Yokomi et al. [26] also employed the DNA barcode region to distinguish *C. pomonella* from native species feeding on cherries in the U.S., along with melt curves and sequencing for identification confirmation. Vulchi et al. [27] developed a DNA high-resolution melt (HRM) curve analysis for discriminating *Amyelois transitella*, *Anarsia lineatella*, *Cadra figullella* and *Plodia interpunctella*, which feed on pistachio and almond in the U.S., but also included *C. pomonella* in their analyses. In Europe, Chen and Dorn [28] developed a restriction fragment length polymorphism (RFLP) assay for discriminating *C. pomonella*, *A. molesta*, *A. funebrana* and *A. lobarzewskii*, which feed internally on *Malus* and *Prunus*. For use in China, Wang et al. [29] developed a real-time PCR assay for identifying *C. pomonella*, *Euzophera pyriella* and *A. molesta*, which all feed on Korla fragrant pears. Despite the availability of these assays, a more comprehensive method of identifying *C. pomonella* from a greater number of species specifically for inspections at U.S. ports of entry is needed. The purpose of this paper is to rapidly identify *C. pomonella* individuals, particularly larvae, among other species often detected at U.S. ports of entry to prevent unnecessary quarantine actions and enhance the focus on discriminating invasive pests not yet established in North America and for which eradication could still be effective.

## 2. Results

### 2.1. Assay Design

DNA barcode sequences were generated for 24 *C. pomonella* and 107 non-target sequences using Sanger sequencing. These sequences were used for confirming the identification of larval specimens, as well as examining species-specific nucleotide differences to determine whether the region could be used for designing primers and probes in a *C. pomonella* identification assay. Although nucleotide sequence differences among species were clear enough to enable molecular identification, this variation was insufficient (based on thermodynamics and position of SNVs) for designing a standard real-time PCR assay. The ITS2 (Internal Transcribed Spacer 2) data (GenBank accessions PQ845417-PQ845427 for those ITS2 sequences generated as part of this study, by contrast, showed sufficient nucleotide differences among species, stretching across indels, that could be used to identify *C. pomonella* in a real-time assay (Supplementary Material S1). Two primer and probe sets were designed within this region and tested using real-time PCR in laboratory analyses. Initial tests showed false-positive results with *C. fagiglandana*, *C. kurokoi,* and *T. leucotreta* with the ITS2-1 primer and probe set, most likely due to intraspecific variation or ribotypic variability within these species. Testing of this primer and probe set was subsequently abandoned. Only the ITS2-4 primer and probe set were considered for analytical sensitivity and specificity.

### 2.2. Analytical Sensitivity

**Figure 1:**
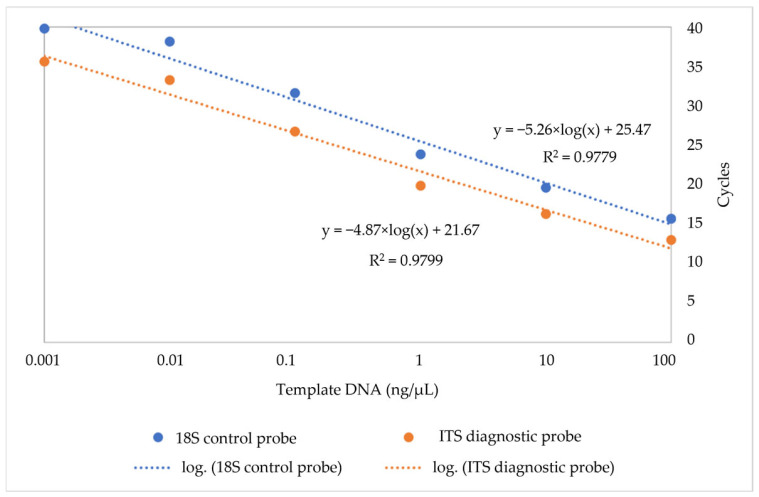
Standard curve of Cq values for serial dilutions of *C. pomonella* for the diagnostic (ITS) probe and control (18S) probes.

Four *C. pomonella* specimens were used to determine the analytical sensitivity of the ITS2 and 18S probes. Both probes were able to detect DNA with concentrations as low as 0.001 ng/µL. The Cq values obtained with the ITS probe and the 18S internal control were similar, with a maximum ΔCq of 4 cycles, although for two *C. pomonella* individuals the ΔCq was as high as 6. DNA concentrations and Cq values were highly correlated for both probes, with R^2^ = 0.98 for both probes (Figure 1). Serial dilution tests showed that, as expected, Cq values of the reaction decreased as DNA template concentrations increased.

### 2.3. Analytical Specificity

**Figure 2:**
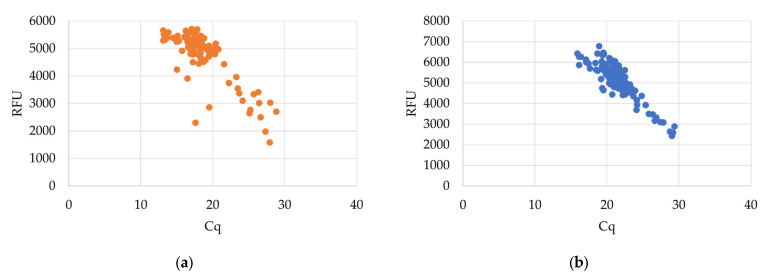
Cq values with their corresponding relative fluorescence units (RFUs) for the (**a**) ITS FAM probe and (**b**) 18S Quasar probe for identification of *C. pomonella.*

**Figure 3:**
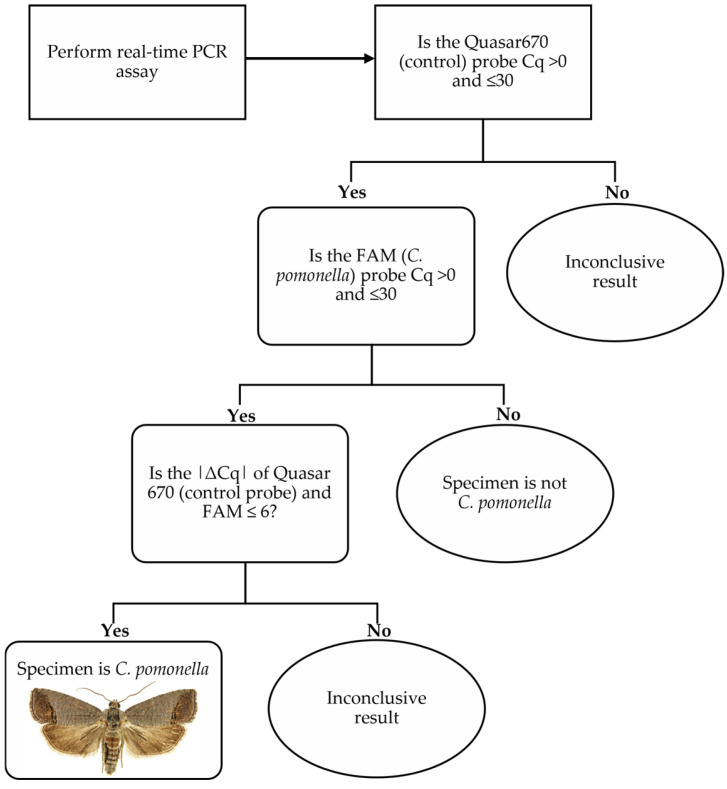
Decision-making tree for identifying *C. pomonella* based on Cq values of the FAM and Quasar probes for the duplex real-time PCR assay.

The developed assay was able to identify all 110 *C. pomonella* specimens, with an average Cq value of 18.7 ± 3.3 for the ITS2 probe and 21.6 ± 2.7 for the 18S internal control probe. Cq values ranged from 13.09 to 28.83 for the ITS2 probe and 15.87 to 29.36 for the 18S probe. Figure 2 compares the Cq values of both probes with their associated relative fluorescence units (RFUs). All non-target specimens produced negative results with the ITS probe, as expected. Analytical specificity was therefore calculated as 100%. Positive results were obtained for all non-targets with the 18S control probe. Cq values averaging 19.8 ± 3.7 were obtained for the 18S probe for non-target individuals. A decision tree outlining the steps necessary for the positive identification of *C. pomonella* based on Cq values of both probes is shown in Figure 3.

## 3. Discussion

The assay developed here enables molecular diagnosis of *C. pomonella* without the need for DNA sequencing. It is also more reliable than current identification assays and specific to larvae intercepted at U.S. ports of entry. We surveyed *C. pomonella* genomes originating from 16 different countries and six continents in our testing, covering most of the geographic range of this cosmopolitan species. Naturalized populations of *C. pomonella* collected in the U.S. were also tested. All target individuals from across this range were positively identified and amplified with both the ITS2 and 18S internal control probes, showing the inclusivity of the primers and probe designed. Assay inclusivity is vital as false negatives resulting from intraspecific variation within *C. pomonella* could result in unnecessary quarantine actions. This assay was designed to discriminate *C. pomonella* from all other related Grapholitini species that are encountered at U.S. ports of entry on similar hosts. For ruling out false positives, the only reliable method is to test the assay against all other similar species that may be encountered on commodities at ports of entry. We tested actual intercepted specimens, including species that were misdiagnosed as *C. pomonella*, species erroneously identified as *C. pomonella*, or tortricid specimens that were only identified at taxonomic levels above species. We also included species that superficially resemble *C. pomonella*, those that are found in similar commodities in the same geographic region as *C. pomonella*, as well as regularly intercepted grapholitines. After testing all these species, the analytical specificity for the assay designed here was 100%. Although it would be impossible to test all species that could be misidentified as *C. pomonella* at ports of entry, by using actual interceptions, and covering the most relevant misdiagnosed species, we can be confident in having a realistic representation of non-target specimens that may be encountered at ports of entry for designing an assay suitable for use for identifying interceptions. It is important to note that the assay was developed only for use on material intercepted at U.S. ports of entry for known tortricid specimens. A relevant issue for *C. pomonella* identification is being able to discriminate this species from other tortricid insects in North America that feed on cherries. This is important for exporting cherries to Japan, where *C. pomonella* is invasive. Since tortricids such as *A. argyrospila*, *A. rosana*, *Argyrotaenia franciscana*, *Epiphyas postvittana*, *Cacoecimorpha pronubana*, *Choristoneura rosaceana*, *Pandemis pyrusana*, and *Spilonota ocellana*, which all feed on cherries in the U.S., were not tested, the assay described here is not suitable for identifying *C. pomonella* in cherry exports without further testing. The assay may be expanded to identify *C. pomonella* domestically if additional native species are tested.

Five different molecular assays for identifying *C. pomonella* for various purposes, including diagnosing the species on cherries designated for export, have been published [7,25,26,27,28,29]. Although the techniques for identification range from diagnostic primers for conventional and real-time PCR, to HRM and RFLP, all previously published assays are based on sequence differences of the 5′ region of the cytochrome c oxidase I gene. For this study, while species-specific diagnostic nucleotides were apparent in this region of the gene, sufficient variation (using thermodynamic criteria) among species to design specific and inclusive primers and hydrolysis probes was not evident. Permissive PCR conditions may result in amplification of specimens differing by as many as three nucleotides in the primer and probe sequences. It is relevant to note that previous assays also evaluated far fewer non-target species, and may not always be reliable since, given the relative lack of nucleotide variation in this region, they may be able to amplify non-target species. For this reason, we concentrated efforts on the ITS region, which has been found to be effective in similar identification assays of other species (e.g., [24,30]). Targeting the ITS region ensured greater reliability than previously published assays as *C. pomonella* could be distinguished from a much larger number of non-target species.

For both probes, DNA concentrations as low as 0.001 ng could be detected. Many molecular assays are reliant on high-quality DNA. Low-quality DNA can prevent or delay amplification or interfere with other enzymatic processes necessary for assay functioning. DNA extracted from intercepted specimens is frequently degraded and of low quality. Larvae from port interceptions are often submitted in 75% ethanol, or other fluids not suitable for preserving DNA, leading to DNA degradation over periods as short as a year [31]. Molecular assays for this purpose therefore have to account for variation in DNA quality. We included DNA from intercepted specimens that had been stored in 70% ethanol for as long as 12 years, as well as recently collected specimens. Despite this range in specimen age, and using intercepted specimens, no non-target specimens failed with the internal control probe, and all *C. pomonella* specimens were identified positively. This analytical specificity is a function of the real-time assay design, which amplifies short DNA fragments and is therefore less sensitive to the effects of DNA degradation that can plague other methods. Additional factors make real-time PCR suitable as a routine diagnostic tool. For instance, the assay is able to yield results in as little as four hours and is therefore suitable for use in instances where identifications are urgently required for quarantine decision-making. Although an overnight DNA extraction period was performed because specimens were used that were several years old, a shorter incubation period is suitable for fresh specimens, such as those usually obtained by port inspectors for routine identifications. The simplicity of the assay allows its implementation in any molecular laboratory equipped with real-time PCR capability.

This study presents a novel and robust molecular approach to identifying *C. pomonella* that is intercepted at U.S. ports of entry. Although the method was tested with larvae and adults, it is likely that similar results will be found with eggs and pupae. Even though *C. pomonella* is not of quarantine significance in the U.S., misdiagnosis of this species or diagnosis only to taxonomic levels above that of species may result in unnecessary quarantine actions. Having a diagnostic assay that can routinely identify *C. pomonella* is useful, particularly with early larval instars that may be difficult to identify since many diagnostic morphological characters are not distinguishable in these specimens. Based on the results obtained, the assay described here is specific and sensitive enough to be fit for its purpose of identifying *C. pomonella* from other insect material intercepted at U.S. ports of entry. As it is, the assay is also suitable for the identification of internal feeding tortricids of *Malus* and *Prunus* in Europe [28]. It can also replace/complement the assay developed by Barcenas et al. [7]. The assay developed here may also be expanded to be suitable for port inspections in other countries.

## 4. Materials and Methods

### 4.1. Insect Material

To ensure applicability to real-world interception scenarios, the bulk of specimens used were obtained from interceptions provisionally identified by port inspectors as Tortricidae and sent to the USDA-ARS Systematic Entomology Laboratory (SEL) for final morphological identification. Larvae and pupae were stored in ethanol while adults were pinned. Additional specimens were collected from pheromone and light traps and obtained from museum collections or donations from collaborators. A list of all specimens included in our analyses is shown in Table 2.

Target specimens of *C. pomonella* from a range of geographic distributions were included to test the inclusivity of the assay. Intercepted larval individuals of *C. pomonella* from Australia, France, Italy, Mexico, Kyrgyzstan, Lebanon, Armenia, Iran, Israel, Czech Republic, Turkey, Peru, and Pakistan were tested as well as donated specimens from South Africa and Argentina. Domestic specimens of *C. pomonella* adults were collected using sticky traps each containing a rubber septum with insect pheromones (Scentry Biologicals Inc, Billings, MT, USA) in Colorado. The specificity of the assay was tested by analyzing non-target species that are taxonomically related to *C. pomonella* or are in interceptions from crops on which *Cydia* species feed. Non-target *Cydia* species that were tested included *C. caryana* from Mexico, *C. fagiglandana* from Morocco, Germany, Italy, and Greece, *C. kurokoi* from South Korea, *C. latiferreana* from Mexico, *C. nigricana* from Nigeria, and *C. splendana* from South Korea and the Netherlands. Additional Grapholitini that were analyzed included *Cryptophlebia ombrodelta*, *Cr*. near *ombrodelta*, *Cr. pallifimbriana*, *Cr. peltastica*, *Ecdytolopha fabivora*, *Fulcrifera fulturana*, *A. packardi*, *A. funebrana*, *A. lobarzewskii*, *A. molesta*, *A. prunivora*, *Gymnandrosoma* sp., *Gy. aurantianum*, *Gy. leucothorax*, *Gy. punctidiscanum*, *Lobesia vanillana*, *Lozotaenia capensana*, *Ofatulena duodecemstriata*, *Pammene fasciata*, *Thaumatotibia batrachopa*, and *T. leucotreta*. Intercepted species that could not be identified to genus using either DNA (due to lack of high confidence matches in databases) or morphology, but could be identified as grapholitines, were also analyzed.

### 4.2. DNA Extraction

DNA extraction was performed using the Lucigen Masterpure Complete DNA and RNA Purification kit (Lucigen, Middleton, WI, USA) according to the manufacturer’s protocols for DNA extraction from tissues, with initial lysis taking place overnight. For adult specimens, a single leg was removed for DNA extractions, while the remaining specimen was retained as a voucher. The leg was macerated using a mini beadbeater and silica-zirconia beads prior to extraction. Larvae, which comprised the bulk of specimens used, were not dissected so that the entire specimen could be retained as a voucher. For this non-destructive DNA extraction protocol entire larval specimens were immersed in the extraction buffer to limit morphological damage to the specimens. After extraction, larval specimens were removed and washed in 100% ethanol before being stored in 100% ethanol as vouchers. Voucher material is stored at the USDA-APHIS-PPQ-S&T laboratory in Fort Collins, CO, USA, or at the Smithsonian Institution.

### 4.3. DNA Barcode Sequencing

DNA barcodes were used to confirm the identity of intercepted or donated specimens, except for some adults, where morphology was sufficient for discriminating species identities. Standard protocols, using the universal primers LepF1/LepR1 [32] and LCO-1490/HCO-2198 [33] were used for both amplification and sequencing. Amplification reactions were performed with TaKaRa Ex Taq HS polymerase (Takara Bio, Shiga, Japan). Thermocycling conditions included initial denaturing at 94 °C for three minutes, followed by 39 cycles of 94 °C for 20 s, 50 °C for 20 s, 72 °C for 30 s, and a final extension at 72 °C for five minutes. PCR amplification was confirmed by gel electrophoresis (Thermo Fisher Scientific, Waltham, MA, USA), after which PCR products were purified using either the QIAquick PCR purification kit (Qiagen, Hilden, Germany) or Exo-SAP-IT PCR Product Cleanup Reagent (Applied Biosystems, Foster City, CA, USA). Sequencing of amplified, purified products was performed in both forward and reverse directions by the University of Chicago Comprehensive Cancer Center Genotyping Facility. Sequences were edited to exclude low-quality calls and consensus sequences generated using Geneious Prime^®^ 2021.0.3. Sequences were identified using the Barcode of Life (BOLD) identification engine with the species-level barcode database. Species identifications were made based on the generated tree and a similarity match of at least 98%.

Since previously published molecular assays for identifying *C. pomonella* were based on the DNA barcode fragment, sequence alignments generated for species identification purposes were also examined for their suitability for designing a real-time identification assay for *C. pomonella*. Criteria used for primer and probe design included a probe sequence with differences of at least 5 consecutive bases, along with at least one primer with at least 3 bp difference to non-target species, along with recommended criteria for real-time PCR primer and probe design [34]. After applying these criteria, no suitable regions for primer and probe design were found in the DNA barcode fragment for unequivocal discrimination of *C. pomonella* from commonly intercepted Grapholitini.

### 4.4. ITS Sequencing

Sequences of the internal transcribed spacer regions (ITS), extending from ribosomal 18S to 28S, or subregions thereof, were obtained from van de Vossenberg et al. [30] as well as publicly available sequences on GenBank, for *C. pomonella*, *C. splendana*, *C. strobilella*, *C. amplana*, *A. molesta*, *A. dimorpha*, *A. funebrana*, and *A. lobarzewskii*. Based on a MAFFT v7.450 [35,36] alignment of these sequences, conducted using Geneious, the ITS2 region was selected as being most suitable for designing primers and probes, using the criteria outlined above, and based on sequence similarity within *C. pomonella* and observed differences from remaining species. Additional sequences from this region were amplified from specimens of *C. kurokoi*, *C. fagiglandana*, *C. latiferreana*, and *A. packardi* using primers ITSF and ITSR [37,38], using a PCR protocol identical to that outlined above for generating mtDNA barcodes. An alignment was generated that included each individual for which ITS sequences were available, which allowed the visualization of regions with interspecific nucleotide differences but little or no intraspecific differences. Two sets of primers and hydrolysis probes, designed across indels (insertion-deletion), were designed for identifying *C. pomonella* (Table 3) using Geneious and Primer3Plus [39] to ensure primer specificity and an optimal assay. OligoCalc version 3.27 was used to calculate the Tm of primers and probes [40]. Primers were purchased from Integrated DNA Technologies (Coralville, IA, USA) while probes were obtained from Biosearch Technologies (Hoddesdon, UK).

### 4.5. Real-Time PCR Assay

In addition to the two sets of primers and probes that were developed for identifying *C. pomonella*, an 18S control probe [41] was also included in the assay to ensure that extracted DNA was suitable for amplification. Initially, the newly designed ITS primer and probe sets were tested in uniplex reactions. After optimization of these primer and probe sets, a multiplex assay was tested that included both the ITS and 18S probes. The multiplex assay was optimized by running probe concentration gradients to determine the optimal amount of each probe, as well as temperature gradients to optimize the annealing temperature. Reaction conditions were optimized to ensure that the ITS2 probe was similar to or less sensitive than the 18S probe to exclude the probability of false negatives. In all runs, a no template control containing water instead of DNA was included. Based on these optimizations, a final protocol was designed that used 10 pmol of each ITS and 18S primer, along with 2 pmol ITS probe, 10 pmol 18S probe, and 2× iTaq Universal Probes Supermix (Bio-Rad Laboratories, Inc., Hercules, CA, USA). All real-time PCR reactions were carried out on a Bio-Rad CFX96 Touch Real-time PCR Detection System (Bio-Rad Laboratories, Inc., Hercules, CA, USA) in 96-well, thin-walled, white-well, hard-shell PCR plates (Bio-Rad Laboratories, Inc., Hercules, CA, USA) that were sealed with optically clear Microseal ‘B’ seals (Bio-Rad Laboratories, Inc. Hercules, CA, USA). After tests to optimize denaturation and annealing temperatures and durations, the PCR program included an initial denaturation at 95 °C for 7.5 min with a ramp rate of 4.4 °C/s, and 39 cycles of 95 °C for 10 s (ramp rate 4.4 °C/s) and 66 °C for 20 s (ramp rate 2.2 °C/s, plate reading,). The plate reading was made after each 66 °C cycle. A lid temperature of 105 °C was maintained throughout all cycles. Baseline settings were computed automatically.

### 4.6. Sensitivity Analysis and Specificity Testing

The sensitivity of the assay was calculated by measuring Cq values of a 10× DNA dilution series from 0.001 ng to 100 ng using four specimens. DNA concentration was determined using a Qubit fluorometer (Invitrogen, Carlsbad, CA, USA). The resulting 24 samples were tested in triplicate for multiplex reactions. Average Cq values at each DNA concentration for each probe were determined and plotted against DNA concentration to determine intercepts.

The specificity of primers and probes for *C. pomonella* was tested using the non-target specimens listed in Table 2.

## Figures and Tables

**Table 1:** Published molecular assays for identifying *C. pomonella*, including the target gene and geographic/species limitations for each assay.

Diagnostic Assay	Locus	Purpose	Geographic Limitations	Species Tested	Reference
Conventional and real-time PCR with diagnostic primers	Cytochrome oxidase I	Differentiating caterpillars feeding internally on pome fruit	North America	*C. pomonella*, *A. molesta*, *A. prunivora*, *A. packardi*	Barcenas et al., 2005 [7]
Restriction fragment length polymorphism (RFLP)	Cytochrome oxidase I	Discriminating internal feeders on *Malus* and *Prunus*	Europe	*C. pomonella*, *A. molesta*, *A. funebrana*, *A. lobarzewskii*	Chen & Dorn, 2009 [28]
DNA barcode sequencing	Cytochrome oxidase I	Identifying internally feeding Lepidoptera in apples	Canada	*C. pomonella*, *A. prunivora*, *A. molesta*	Firlej et al., 2013 [18]
Conventional PCR with diagnostic primers	Cytochrome oxidase I	Discriminating *C. pomonella* from other tortricid pests of cherries	North America	*Archips argyrospila*, *A. rosana*, *Argyrotaenia franciscana*, *Cacoecimorpha pronubana*, *Choristoneura rosaceana*, *Epiphyas postvittana*, *A. molesta*, *A. packardi*, *A. prunivora*, *Pandemis pyrusana*, *Spilonota ocellana*	Amano & Higo, 2015 [25]
Real-time PCR	Cytochrome oxidase I	Identification of pests of Korla fragrant pears	China	*C. pomonella*, *Euzophera pyriella*, *A. molesta*	Wang et al., 2018 [29]
High resolution melt (HRM) curve analysis	Cytochrome oxidase I	Identification of lepidopteran pests of almonds and pistachios	North America	*Amyelois transitella*, *Anarsia lineatella*, *A. molesta*, *Choristoneura rosaceana*, *Cadra figulilella*, *Plodia interpunctella. C. pomonella* and Pyralidae, Noctuidae and Crambidae collected in almond orchards were also included	Vulchi et al., 2021 [27]
Conventional multiplex PCR with diagnostic primers	Cytochrome oxidase I	Discriminating tortricid pests feeding internally on pome fruit	North America	*C. pomonella*, *C. latiferreana*, *A. molesta*, *A. prunivora*, *A. packardi*	Yokomi et al., 2022 [26]

**Table 2:** Specimens used for testing the inclusivity and specificity of a real-time PCR assay for identifying *C. pomonella.*

Species	Geographic Origin	Host	Collection Method	Life Stage	Number of Specimens
*Aspila funebrana*	Azerbaijan	*Prunus* sp.	Interception	Larva	3
	Pakistan	*Prunus domestica*	Interception	Larva	3
	Serbia	*Prunus domestica*	Interception	Larva	6
*Aspila lobarzewskii*	United Kingdom	*Malus domestica*	Interception	Larva	1
*Aspila molesta*	Germany	*Cydonia oblonga*	Interception	Larva	1
	Albania	Unknown	Interception	Larva	1
*Aspila packardi*	Mexico	*Cydonia oblonga*	Interception	Larva	2
*Aspila prunivora*	USA, California	Unknown	Museum Specimen	Adult	1
*Cryptophlebia pallifimbriana*	Tahiti	Unknown seeds	Interception	Larva	1
	Ghana	*Capsicum* sp.	Interception	Larva	1
*Cryptophlebia peltastica*	South Africa		Laboratory colony	Adult	3
*Cryptophlebia* sp. near *ombrodelta*	Bangladesh	*Litchi chinensis*	Interception	Larva	2
	Hawaii	*Litchi chinensis*, *Pithecellobium dulce*	Interception	Larva	3
	Philippines	*Pithecellobium* sp., *P. dulce*	Interception	Larva	5
	India	*Tamarindus indica*, Unknown	Interception	Larva, pupa	3
	Myanmar	Baggage	Interception	Larva	1
*Cydia caryana*	Mexico	*Carya illinoinensis*	Interception	Larva	1
*Cydia fagiglandana*	Morocco	Unknown	Interception	Larva	1
	Germany	Unknown	Interception	Larva	1
	Italy	*Castanea sativa*	Interception	Larva	1
	Greece	*Castanea sativa*	Interception	Larva	1
*Cydia kurokoi*	Unknown	Unknown	Interception	Larva	2
	South Korea	*Castanea* sp.	Interception	Larva	12
	Unknown	*Castanea* sp.	Interception	Larva	4
*Cydia latiferreana*	Mexico	Unknown, *Citrus sinensis*, *Quercus* sp., *Pinus* sp.	Interception	Larva	13
*Cydia nigricana*	Nigeria	Unknown	Interception	Larva	1
*Cydia pomonella*	Argentina	*Pyrus* sp.	Donation	Larva	6
	Armenia	*Prunus armeniaca*	Interception	Larva	3
	Australia	*Citrus* sp.	Interception	Adult	1
	Czech Republic	*Malus* sp.	Interception	Larva	1
	France	Unknown, *Malus domestica*	Interception	Larva	3
	Iran	*Malus domestica*	Interception	Larva	1
	Israel	*Malus* sp.	Interception	Larva	2
	Italy	*Juglans* sp.	Interception	Larva	1
	Kyrgyzstan	*Malus* sp.	Interception	Larva	1
	Lebanon	*Pyrus* sp.	Interception	Larva	1
	Mexico	*Zea mays*, *Capsicum annuum*, *Malus* sp., *Cydonia* sp.	Interception	Larva	7
	Pakistan	*Malus domestica*	Interception	Larva	1
	Peru	*Cydonia oblonga*	Interception	Larva	1
	South Africa	Unknown	Donation	Larva	10
	Turkey	*Malus* sp.	Interception	Larva	1
	Unknown	Unknown	Interception	Larva	1
	USA	Unknown	Donation	Adult	1
	USA, California	Unknown	Museum specimen	Adult	1
	USA, Colorado	Unknown	Museum specimens, field collections	Adult	69
*Cydia splendana*	South Korea	*Castanea* sp.	Interception	Larva	1
	Netherlands	*Castanea sativa*	Interception	Larva	2
*Ecdytolopha fabivora*	El Salvador	Unknown	Interception	Larva	1
*Fulcrifera fulturana*	Bangladesh	*Syzygium jambos*	Interception	Larva	1
*Gymnandrosoma* *aurantianum*	Puerto Rico	*Mangifera indica*	Interception	Larva	2
	Mexico	*Byrsonima crassifolia*	Interception	Larva	1
*Gymnandrosoma leucothorax*	Unknown	*Psidium guajava*	Interception	Larva	1
*Gymnandrosoma punctidiscanum*	Unknown	Unknown	Interception	Larva	1
*Gymnandrosoma* sp.	Peru	*Theobroma cacao*	Interception	Larva	1
	Mexico	*Byrsonima crassifolia*, *Pithecellobium dulce*, *Litchi chinensis*	Interception	Larva	3
*Lobesia vanillana*	Kenya	Unknown	Light Trap	Adult	2
*Lozotaenia capensana*	South Africa	Unknown	Light Trap	Adult	2
*Ofatulena duodecemstriata*	Mexico	*Pithecellobium dulce*	Interception	Larva	3
*Pammene fasciata*	Greece	*Castanea sativa*	Interception	Larva	1
	Morocco	*Quercus lusitanica*	Interception	Larva	1
*Thaumatotibia batrachopa*	South Africa	*Macadamia* sp.	Field Collection	Larva	3
*Thaumatotibia leucotreta*	Cape Verde	*Ziziphus jujuba*, *Sansevieria trifasciata*	Interception	Larva	5
	Malawi	*Zea mays*	Interception	Larva	1
	Nigeria	*Psidium* sp., *Cola* sp.	Interception	Larva	2
	Ghana	*Capsicum* sp.	Interception	Larva	3
	Kenya	*Macadamia* sp.	Interception	Larva	1
	Somalia	*Psidium guajava*	Interception	Larva	1
Tortricidae	Brazil	*Araucaria angustifolia*	Interception	Larva	1

**Table 3:** Primers and hydrolysis probes designed for a real-time PCR identification assay for *C. pomonella.*

Oligo Name	Nucleotide Sequence	Amplicon Length (bp)	Oligo Length (bp)	%GC	Tm * (°C)
*Cydia pomonella* primer/probe set 1 (discarded due to lack of specificity)					
Cpom1F	5′-GGGCCGGCTGCATAAATA	109	18	55.6	56.3
Cpom1R	5′-GACGCACGAAATCAACAAC		19	47.4	55
CpomP1	5′-FAM-TGTACGCTATTGAGGGTTCGTTTGAAGA-BHQ-2		28	43	67.2
*Cydia pomonella* primer/probe set 2					
Cpom4F	5′-CCCGCGTGTGTGTAATAAAT	78	20	45	56.4
Cpom4R	5′-CGACGACGACAACGACAA		18	55.6	56.3
CpomP4	5′-FAM-TCATCATTGTACACGTATCGTGTTMCAGCT-BHQ-2		30	43.3	68–69
Control primer and probe set [39]					
RT-18S-F2	5′-ACCGCCCTAGTTCTAACCGTAAA	68	23	48	62.9
RT-18S-R2	5′-CCGCCGAGCCATTGTAGTAA		20	55	60.5
RT-18S-P2	5′-Quasar670-TGTCATCTAGCGATCCGCCGA-BHQ-2		21	57	63.2

* Calculated using OligoCalc version 3.27 [40].

## Data Availability

All data not included in public repositories is available upon request from the corresponding authors.

## Supplementary Materials

The following supporting information can be downloaded at: https://www.mdpi.com/article/10.3390/ijms26020707/s1.
