# Supplementary material for: A Real-Time PCR Assay for Detecting Codling Moth Cydia pomonella on Material Intercepted at U.S. Ports of Entry—A Valuable Tool for Specimen Identification
Source: Int J Mol Sci. 2025 Jan 15;26(2):707. doi: 10.3390/ijms26020707 (PMC11766013; doi:10.3390/ijms26020707)
Supplement: Supplementary file 1 [file ijms-26-00707-s001.zip › ijms-3390339-supplementary.pdf]

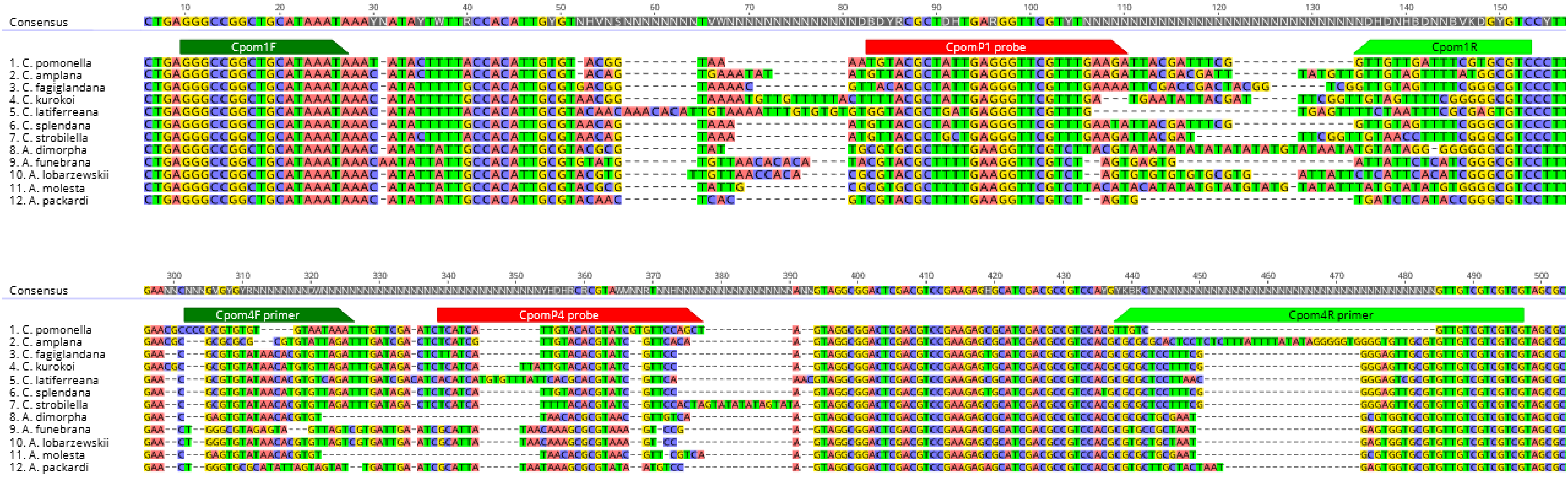

Supplementary Figure S1. Alignments of 12 tortricid ITS2 regions to highlight differences present within the primer and probe binding sites between target *C. pomonella* and closely related non-target species.
